# Prevalence and associated factors of diabetes mellitus among Governmental Civil Servants at Guji Zone, Oromia Region, Ethiopia, 2021. A community-based cross-sectional study

**DOI:** 10.1371/journal.pone.0267231

**Published:** 2022-04-15

**Authors:** Takala Utura, Anteneh Fikrie

**Affiliations:** School of Public Health, Institute of Health, Bule Hora University, Bule Hora, Ethiopia; Government College University Faisalabad, Pakistan, PAKISTAN

## Abstract

**Background:**

Despite it being easily preventable, still diabetes mellitus is found in every population in the world and all regions, with the greatest escalation in low and middle-income countries. Moreover, undiagnosed or poorly controlled diabetes can lead to lower limb amputation, blindness, and kidney disease. However, there is a paucity of information on the magnitude and associated factors among adult populations in rural pastoral areas. Therefore, this study aimed to assess the magnitude of diabetes mellitus and associated factors among Guji Zone Government Civil Servants, Southern Ethiopia.

**Methods:**

Cross-sectional study was conducted from March 1-14/2018, among 437 randomly selected Government employees of Guji Zone. A self-administered questionnaire was used to collect data. Data were coded and entered using Epi-data version 3.1 and exported to SPSS version 20 for analysis. Multivariable logistic regression analysis was done to identify significant factors associated with the magnitude of DM. P<0.05 was used to declare statistical significance and odds ratio with 95% confidence interval were calculated.

**Result:**

The median (±IQR) age of participants was 33 (±14) years of age. Overall, the prevalence of DM in the study population was found to be 16 (3.9%) [95% CI: 2.2–5.6%]. The prevalence of DM among males and females was 3.8% and 4.2% respectively. Age (<35 years) [0.21 (0.04–0.94)], increasing salt amount in dietary feeding [14.31(1.28–159.2)], Consumption of vegetable &fruit once per week [23.38(2.01–269.17)], diagnosed with HTN [21.35(2.28–199.37)], and Family history of DM [9.42(1.72–51.42)] were significantly associated with DM.

**Conclusion:**

Comparably lower prevalence of previously undiagnosed DM was found by this study. Being old, excess salt consumption, intake of vegetables & fruit once per week, hypertension, and family history of DM were significantly associated with DM. Therefore, the zonal Health department should enhance and strengthen the provision of health education programs and counseling about nutrition, weight control, and appropriate physical activity and advised the communities for mass screening for diabetes.

## Background

Diabetes Mellitus (DM) is one of the four priority non-communicable diseases (NCDs) identified by the World Health Organization (WHO) [1]. It is a common, chronic, costly, and metabolic disease and characterized by high levels of glucose in the blood, which results from lack of insulin (type 1 diabetes), or insufficient insulin and insulin resistance (type 2 diabetes) [2]. Type-2 diabetes is the most common is usually in adults, which occurs when the body becomes resistant to insulin or doesn’t produce enough insulin by the pancreas and accounts for nearly 95% of all diabetes cases globally [3]. Evidence exists that type 2 diabetes can be prevented or delayed, and there is accumulating evidence that remission of type 2 diabetes may sometimes be possible [4]. Diabetes should be diagnosed if one or more of the following criteria are met if fasting plasma glucose ≥7.0mmol/L (126mg/dl) or two-hour plasma glucose after 75g oral glucose load (oral glucose tolerance test (OGTT)) is ≥11.1mmol/L (200mg/dl) or HbA1c Random plasma glucose in the presence of symptoms of hyperglycemia is ≥11.1mmol/L (200mg/dl) [3].

Diabetes is a major health issue that has reached alarming levels. Currently, more than half a billion people are living with diabetes globally [2]. The highest prevalence (75%) was observed in low and middle-income countries. Globally, one in two people living with diabetes is undiagnosed [5]. The majority, 70% of patients with undiagnosed diabetes are found in sub-Saharan Africa. Similarly, in the country, the numbers of undiagnosed diabetes cases are expected to exceed 66,000. Similarly, According to the IDA estimate of 2017, Ethiopia is the leading country among the top five high burden African countries for the number of people with diabetes [6].

DM was recognized as the seventh leading cause of death in 2016 globally [3]. Moreover, it has been reported as a major cause of premature dying, blindness, kidney failure, heart attack, stroke, and lower limb amputation [3,6–9]. By 2040, One in three people with diabetes will develop diabetic retinopathy which can cause blindness [5]. Direly the undiagnosed, untreated or poorly controlled diabetes can cause devastating, irreversible complications such as visual impairment and blindness, kidney failure, heart attack, stroke, lower limb amputation, and erectile dysfunction [1]. DM is not merely affect the health of individuals but also it has a direct impact on the healthcare costs crisis, loss of labor productivity, and decreased rates of economic growth [3,5]. Evidence showed that nearly 12% of global health expenditure is spent on diabetes [1,10]. In 2021, 65.3 billion USD was spent on diabetes in the South and Central America Region, representing 6.7% of the total spent worldwide [2]. Similarly, the American Diabetes Association (ADA) estimated the national costs of diabetes in the USA to be 192 billion dollars in 2020 [5].

Different studies have identified different risk factors for the occurrence of DM. The most commons are; poor eating and dietary habits, tobacco use, physical inactivity, overweight and obesity, excessive consumption of alcohol, old age, and hypercholesterolemia [11–13]. In Ethiopia, findings from the Global Burden of Disease study 2013 found that the proportion of NCD deaths associated with low fruit consumption slightly increased (11.3% in 1990 and 11.9% in 2013) [14]. Early diagnosis and appropriate treatment and access to effective services are keys to preventing the costly end-stage complications of diabetes and achieving healthy outcomes [1,3,4]. Lifestyle interventions and socially responsible policies and market interventions within and beyond the health sector can promote healthy nutrition and physical activity and prevent diabetes [1].

To our knowledge, there was no prior study that has been conducted in the current study area. Moreover, the information which quantifies the undiagnosed DM level particularly among the government civil servants in urban pastoralist areas is essential to verify the extent of the problem. Therefore, this study aimed to assess the magnitude of undiagnosed diabetes mellitus and associated factors among Guji Zone Government Civil Servants, Oromia, Southern Ethiopia.

## Methods

### Study setting, design, and period

A community-based cross-sectional study was conducted from March, 1 to 14, 2021 among civil servants aged above 18 years in Guji Zone, Oromia, Southern Ethiopia. The zone is located around 598 km far from Addis Ababa, the capital city of Ethiopia. According to the Zone, public service office report of 2019 the total civil servants working at zonal level sectors were estimated to be 1247, of which 754 are men whereas 493 are women.

### Population, sample size determination, and sampling procedure

All government employees found in the Guji Zonal level were our source population. Whereas, all randomly selected government employees were our study populations. Contract staff and staff having less than 6 months’ work experience were excluded. The sample size was determined using the single population proportion formula with the assumptions of Proportion of DM (6.5%) among Individuals Aged ≥15 years in Mizan-Aman Town, Southwest Ethiopia [15], 95% Confidence interval [Z = 1.96), 2% margin of error, and 10% non-response rate for the compensation of the potential non-responses. Then, the minimum calculated sample size became 642. However, the total number of the governmental employee at the Guji Zone level was less than 10,000 (N = 1247), so we considered the population correction formula to get the appropriate representative sample size. Consequently, the final sample size after correction became 437.

A simple random sampling technique was employed to select the study participants. First, all the 32 sectors found at Guji Zone were categorized into four strata’s namely: economic sectors (175 employees), administration and political sectors (206 employees), social sectors (559 employees), and urban coordination sectors (307 employees), and then the lists of employees in each stratum were obtained from Guji Zone public service office. Then, the sample size was distributed to each stratum based on population sizes. Finally, a simple random sampling technique using computer-generated random numbers was used to select the study subjects.

### Data collection tools, procedures, and quality control

A self-administered questionnaire was used to collect data. The questions used to assess practice were adapted from the WHO STEP wise approach to chronic disease risk factor surveillance instrument [16] and Ethiopian national NCD guideline [11]. The questionnaire was primarily prepared in English version then translated into the local language; ‘Afaan Oromoo’ then it was translated back into the English version to check the consistency of the data. Four laboratory technicians and two health officers’ were recruited as data collectors and supervisors respectively. The one-day training was given to data collectors, and supervisors on the details of the instruments, data collection procedure, supervision and communication. A pre-test was conducted on 5% (22) individuals of the total sample size in Liban district government employees, two weeks before actual data collection. Supervisors have checked all procedures and completeness of formats randomly on every each single day of data collection. Furthermore, the data were entered by using Epi-Data to keep and control the quality of data.

Physical activity was measured by using the WHO physical activity questionnaire [16]. The questionnaire assessed work-related activity, walking, sport, and recreational activity, and time spent sitting per day. Work-related activities were categorized as work involving vigorous intensity and moderate-intensity activities. Work involving vigorous-intensity activity was measured by asking the question ‘Does your work involve vigorous intensity activity that causes large increases in breathing or heart rate like (*carrying or lifting heavy loads*, *digging or construction work*) for at least 10 minutes continuously?’, and work involving moderate-intensity activity was measured by asking the question ‘Does your work involve moderate-intensity activity that causes small increases in breathing or heart rate such as brisk walking (*or carrying light loads*) for at least 10 minutes continuously?’ Based on this, an adult person should do at least 150 minutes of moderate-intensity work or 75 minutes of vigorous-intensity work, or 60 minutes of a combination of vigorous and moderate-intensity work per week. If the self-reported physical activity did not fit with the WHO recommendation, participants were categorized as physically inactive [17].

### Measurements

The respondents’ weight and height were measured using standard equipment. Height was measured by sliding meter and read in centimeters to the nearest 0.1 cm and recorded. Weight was measured using a digital weighing scale while the participant was wearing light clothes and recorded in kilograms to the nearest 0.1 kg. The blood pressure (BP) was taken by mercury sphygmomanometer (appropriate cuff size) in the sitting position where the participant’s arm was maintained at the level of the heart. The BP was measured twice at least 5 minutes apart for each participant [5]. Fasting blood glucose level was measured using a glucometer. Fasting blood glucose level was taken was measured after the study participants fasted overnight for at least 12 hours [17–19].

### Operational definitions

- **Administration and political cluster sectors:** administration, political office, security, police, court, and justice departments were included. **Economy cluster sectors:** Finance, finance plan commission, Bank, Pastoral office, disaster preparedness, and prevention office, cooperative work association, irrigation, rural land management, water and energy departments were included. **Social cluster sectors: H**ealth, women, and child affairs, culture and tourism, social issue, youth and sport, Education Departments were included under this sector. **Urban coordination cluster sectors:** entrepreneurship, urban land management, urban administration, income revenue, technical and vocational, investment, construction, government communication, plan and budget, trade, and transport departments were included.
- Diabetes mellitus was defined as FBS level ≥126 mg/dl on two separate measurements or a self-report of the previous diagnosis by healthcare providers or receiving diabetes treatment. Whereas, undiagnostic diabetes was defined as participants whose FBS level was ≥126 mg/dl on two separate measurements and were unaware of it before the survey [17,19].
- Hypertension was defined as sustained high BP (systolic BP≥ 140 or diastolic BP≥ 90 mmHg) or reported regular use of antihypertensive medication [17,19,20]
- Body Mass Index (BMI) was calculated based on weight in kilogram (kg) and height in squared meters (m^2^). The BMI was categorized as underweight (<18.5 kg/m^2^), normal (18.5 to 24.9 kg/m^2^), overweight (25 to 29.9 kg/m^2^) and obese (>30 kg/m^2^) [17,20].
- Current tobacco smokers were defined as using tobacco products and alcohol within the preceding month before the study [17].

### Data management, processing, and analysis

The collected data were checked for completeness and consistency, then entered into Epi data version 3.1 and exported to SPSS version 21 for analysis. Descriptive statistics like frequency and percentage were computed and presented using statistical tables. Bi-variable and multivariable binary logistic regression was used to identify associated factors of DM. Variables with a p-value <0.25 during the bi-variable logistic regression were identified as candidate variables for the final model, multivariate logistic regression to control the potential effect of confounding variables. Hosmer- Lemeshow goodness of fit was used to test the fitness of the model. Adjusted odds ratio (AOR) with 95% confidence intervals (CIs) was used to declare the statistical significances.

### Ethical consideration

A primary ethical approval letter was obtained from Bule Hora University Institutional Review Board (IRB). Then additional support letter was secured from Guji Zone Administration Office which helped us to get from each study sector. Data collection was continued after the purposes of the study were explained and verbal informed consent was obtained from each participant. To keep confidentiality the participants never write their name on questionnaires.

## Results

### Socio-demographic variables

A total of, 410 governmental workers were included and make a response rate of 93.8%. Three-fourths of the respondents were married. The median (±IQR) age of participants was 33 (±14) years of age and the majority144 (35.1%) were found in the age group of < 30 years. Regarding the educational status of the study participants, 281 (68.5%) had a first degree and above educational status (Table 1).

**Table 1:** Socio-demographic characteristics of the governmental employees in East Guji Zone, Southern Ethiopia, 2021.

Variables	Category	Frequency	Percentage (%)
Sex of respondent	Male	292	71.2
	Female	118	28.8
Age (years)	<30	144	35.1
	30–40	130	31.7
	40–50	85	20.7
	≥50	51	12.6
Marital status	Married	315	76.8
	Single	12	2.9
	Divorced/widowed	83	20.3
Educational status	Certificate & below	49	12
	Diploma	80	19.5
	Degree & above	281	68.5
Profession	Health professional	33	8
	Non-Health professional	377	92

### Behavioral characteristics of study participants

Almost one-in-twenty, 4.6% and 4.1% of the participants reported that they had ever smoked a cigarette and currently smoke cigarettes respectively. Pertaining to the participant’s alcohol consumption status, around three-in-eight 150 (36.6%) of the participants ever consumed alcohol. Of the total participants who consumed alcohol, 12 (8%) were consumed more than five days per week in the last 12 months. Overall, more than quarters, 119 (29%) of the participants who consumed alcohol were heavy drinkers (Table 2).

**Table 2:** Behavioral characteristics of the governmental employees in East Guji Zone, Southern Ethiopia, 2021.

Variables	Category	Frequency	Percent (%)
Have you ever smoke cigarette	Yes	19	4.6
	No	391	95.4
Currently do you smoke cigarette	Yes	17	4.1
	No	393	95.9
Ever consumed an alcohol	Yes	150	36.6
	No	260	63.4
In the last 12 months how many days per week did you consumed (n = 150)	≥ 5days/week	12	8
	1–4 days/week	34	22.7
	2–3 days/month	60	40
	Once monthly	44	29.3
How many drinks containing alcohol do you have on a typical day? (n = 150)	1–2	70	46.7
	3–4	55	36.6
	5–6	19	12.7
	7–9	2	1.3
	≥ 10	4	2.7
How often do you have six or more drinks on one occasion in the last one month?	Never	294	71
	One time	34	8.3
	Two times	29	7.1
	Three-to-four times	34	8.3
	More than five times	22	5.4
Alcohol consumption status	Light	291	71
	Heavy drinker	119	29
Do you chew chat	No	338	82.4
	Yes	72	17.6
Do you chew chat daily?	No	53	73.6
	Yes	19	24.4

### Physical activity and dietary patterns characteristics of the participants

Among all the study participants, nearly four-in-five 322 (78.5%) had consumed vegetable oil. More than three-in-five 258 (62.9%) of the study participants increase the salt amount in their diets. Similarly, three-in-five, 245 (59.8%) of the study participants were consumed fruits and vegetables. Of the study participants who were consumed fruits and vegetables, about 32.2% and 54.1%, consumed vegetables and fruit three times per week and less than five servings of fruits and vegetables per day respectively. Nearly two-third 265 (64.6%) of the study participants were involved in work mostly in sitting or standing with walking for no more than 10 minutes at a time. Whereas, more than two-in-seven 126 (30.67%) of the participants were involved in work having vigorous activity, like [*heavy lifting*, *digging or construction work*] for at least 10 minutes at a time. Overall less than one-third, 116 (28.3%) of the participants had active physical activity (Table 3).

**Table 3:** Physical activity and dietary patterns characteristics of the governmental employees in East Guji Zone, Southern Ethiopia, 2021.

Variables	Category	Frequency	Percent (%)
Oil consumed	Non- vegetable oil	88	21.5
	Vegetable oil	322	78.5
Excess salt consumption	Yes	258	62.9
	No	152	37.1
Consumption of fruit and vegetables	No	165	40.2
	Yes	245	59.8
Consumption of vegetable and fruit/week	Daily	22	9
	Three times	79	32.2
	Twice	93	38
	Once weekly	51	20.8
Vegetables and fruits serving in a single day	1–5	222	54.1
	≥ 5	23	5.6
	Never	165	40.2
Does your work involve mostly sitting or standing with walking for no more than 10 minutes at a time	No	145	35.4
	Yes	265	64.6
Does your work involve vigorous activity, like [*heavy lifting*, *digging or construction work*] for at least 10 minutes at a time	No	284	69.3
	Yes	126	30.7
Number of vigorous activities/week	Once	38	9.3
	Twice	36	8.8
	≥ three times	52	12.7
	Never	284	69.3
Do you walk or use a bicycle (*pedal cycle*) for at least 10 minutes continuously?	No	378	92.2
	Yes	22	7.8
In a typical week, on how many days do you walk or bicycle for at least 10 minutes?	<2 days/week	393	95.9
	≥ 2 days/week	17	4.1
Do you work different physical activity during recreational time?	No	282	68.8
	Yes	128	31.2
Physical activity	Inactive	294	71.7
	Active	116	28.3

### Clinical characteristics of study participants

Of the total participants, one in ten 40 (9.8%) and one in five had a family history of DM and HTN respectively. One-in-six, 67 (16.3%) of the study participants had a diagnosis of hypertension. The mean (±SD) weight in kilogram and the mean (±SD) height in a meter of the participants were found to be 71.24 (±12.69) kg and 1.70 (±0.08) meters respectively. On the other hand, the mean (±SD) BMI of the study participants was 24.41 (±4.02) Kg/M^2^. Overall, around 136 (33.2%) and 36 (8.8%) of the study participants were found to be overweight and obese respectively (Table 4).

**Table 4:** Clinical characteristics of the governmental employees at East Guji Zone, Southern Ethiopia, 2021.

Variables	Category	Frequency	Percent (%)
Family history of DM	No	370	90.2
	Yes	40	9.8
Family history of Hypertension (HTN)	No	323	78.8
	Yes	87	21.2
Do you have HTN	No	343	83.7
	Yes	67	16.3
Did you ever checked up for any Non Communicable Disease	**No**	263	64.1
	**Yes**	147	35.9
Did you checked within last one year	**No**	54	36.7
	**Yes**	93	63.3
Body mass index	Underweight	13	3.2
	Normal	225	54.9
	Overweight	136	33.2
	Obese	36	8.8

### The prevalence of diabetes mellitus

Overall, the prevalence of DM in the study population was found to be 16 (3.9%) [95% CI: 2.2–5.6%]. The prevalence of DM among males and females was 3.8% and 4.2% respectively. The prevalence of diabetes mellitus among the participants who have a family history of DM was found to be 17.5%, which is higher than any other group. On the other hand, the second 13.6% and third 10.5% higher prevalence of diabetes mellitus were found among participants who have been diagnosed with HTN and who have ever smoked cigarettes respectively.

### Factors associated with diabetes mellitus among governmental employees

During the bivariable logistic analysis variables like age, increasing salt amount in feeding, Consumption of vegetable &fruit/week, diagnosed with HTN, Family history of DM, BMI, and ever checked for Non-Communicable Diseases were found to be significantly associated with diabetes mellitus at a p-value of <0.25. However, after controlling the potential confounding variables by the multivariable logistic analysis: Age (<35 years) [0.21 (0.04–0.94)], increasing salt amount in dietary feeding [14.31(1.28–159.2)], Consumption of vegetable &fruit once per week [23.38(2.01–269.17)], diagnosed with HTN [21.35(2.28–199.37)], and Family history of DM [9.42(1.72–51.42)] were significantly associated with DM.

Accordingly, participants who are <35 years of age were 79% less likely of contracting DM as compared to participants with the age of ≥35 years [0.21 (0.04–0.94)]. Similarly, participants who were consumed excess salt were fourteen times more likely to experience DM as compared to their counterparts [14.31(1.28–159.2)]. Study participants who had consumed vegetables and fruits once per week were twenty-three times more likely of developing DM as compared to participants who had consumed vegetables and fruits more than three times per week. On the other hand, participants who have hypertension were twenty-nine times more likely of having DM compared to their counterparts [21.35(2.28–199.37)]. Lastly, participants who had a family history of DM were nine times more chance of contracting DM compared to their counterparts [9.42(1.72–51.42)] (Table 5).

**Table 5:** Factors associated with magnitude of DM among the governmental employees at East Guji Zone, Southern Ethiopia, 2021.

	Variable	DM		COR (95%CI)	AOR (95%CI)
		Yes N (%)	No N (%)		
Age (years)					
	<35	3 (1.3)	223 (98.7)	0.17 (0.05–0.63)	0.21 (0.04–0.94)**
	≥35	13 (7.1)	171 (92.9)	1	1
Sex					
	Male	11(3.8)	281 (96.2)	0.88 (0.30–2.60)	-
	Female	5 (4.2)	113 (95.8)	1	-
Marital status					
	Married	13 (4.1)	302 (95.9)	1.32 (0.36–4.73)	-
	Others*	3 (3.2)	92 (96.8)	1	-
Educational status					
	Diploma & below	6 (4.7)	123 (95.3)	1.32 (0.37–3.71)	-
	Degree & above	10 (3.6)	271 (96.4)	1	-
Ever smoke cigarette					
	Yes	2 (10.5)	17 (89.5)	3.16(0.66–15.06)	-
	No	14 (3.6)	377 (96.4)	1	-
Ever consumed an alcohol					
	Yes	9 (6)	142 (94)	2.28(0.83–6.25)	-
	No	7 (2.7)	252 (97.3)	1	-
Alcohol consumption status					
	Heavy	9 (7.6)	110 (92.4)	3.31(1.20–9.13)	-
	Light	7 (2.4)	284 (97.6)	1	-
Chew chat					
	Yes	2 (2.8)	70 (97.2)	0.66(0.14–2.97)	-
	No	14 (4.1)	324 (95.9)	1	-
Type of Oil consumption					
	Vegetable oil	14 (4.3)	308 (95.7)	1.95(0.43–8.76)	-
	Non-vegetable oil	2 (2.3)	86 (97.7)	1	-
Excess salt consumption					
	Yes	13(5)	245 (95)	2.63(0.73–9.40)	14.31(1.28–159.2)*
	No	3 (2)	149 (98)	1	1
Consumed fruit & vegetables					
	Yes	13 (5.3)	232 (94.7)	3.02(0.84–10.78)	-
	No	3 (1.8)	162 (98.2)	1	-
Consumption of vegetable &fruit/week					
	Once weekly	5 (9.8)	46 (90.2)	5.38(1.01–28.77)	23.38(2.01–269.17)*
	Twice weekly	6 (6.5)	87 (93.5)	3.41(0.67–17.35)	7.36(0.94–57.60)
	≥ Three times weekly	2 (2)	99 (99)	1	1
Working involves sitting or standing & walking for no more than 10 minutes					
	Yes	13 (4.9)	252 (95.1)	0.41(0.11–1.46)	0.45(0.07–2.57)
	No	3 (2.1)	142 (97.9)	1	1
Working involves in vigorous activity					
	Yes	5 (4)	121 (96)	0.97(0.33–2.86)	-
	No	11 (3.9)	273 (94.1)	1	-
Working different physical activity during recreational time					
	Yes	3 (2.3)	125 (97.7)	0.49(0.13–1.77)	-
	No	13 (4.6)	269 (95.4)	1	-
Vigorous activity category					
	Active	2 (2.9)	68 (97.1)	0.68(0.15–3.08)	-
	Inactive	14 (4.1)	326 (95.9)	1	-
Moderate physical activity					
	Active	2 (3)	65 (97)	0.72(0.16–3.25)	-
	Inactive	14 (4.1)	329 (95.9)	1	-
Physical activity					
	Active	3 (2.6)	113 (97.4)	0.57(0.16–2.05)	-
	Inactive	13 (4.4)	281 (95.6)	1	-
Family history of HTN					
	Yes	2 (2.3)	85 (97.7)	0.51(0.11–2.33)	-
	No	14 (4.3)	309 (95.7)	1	-
Diagnosed with HTN					
	Yes	9 (13.6)	57 (86.4)	7.60(2.72–21.22)	21.35(2.28–199.37)**
	No	7 (2)	337 (98)	1	1
Family history of DM					
	Yes	7 (17.5)	33 (82.5)	8.50(2.97–24.31)	9.42(1.72–51.42)**
	No	9 (2.4)	361 (97.6)	1	1
Body Mass Index (BMI)					
	<25Kg/m2	5 (2.1)	233 (97.9)	0.31(0.10–0.92)	0.79(0.15–4.21)
	≥25Kg/m2	11 (6.4)	161 (93.6)	1	1
Ever checked for NCD					
	Yes	10 (6.8)	137 (93.2)	3.12(1.11–8.78)	0.23(0.031–1.73)
	No	6 (2.3)	257 (97.7)	1	1
NCD checked within last one year					
	Yes	7 (7.5)	86 (92.5)	1.38(0.34–5.59)	-
	No	3 (2.3)	51 (97.7)	1	-

*p-value<0.05

**p-value<0.001.

## Discussion

This study aimed to assess the prevalence and associated factors of Diabetes Mellitus among Government Civil Servants in Guji Zone, Oromia Region, Ethiopia, 2021. According to the study result, the overall prevalence of previously undiagnosed DM was found to be 3.9% [95% CI: 2.2–5.6%]. On the other hand, after controlling for the potential confounding variables by the multivariable logistic regression analysis: Age (<35 years), excess salt consumption, intake of vegetables & fruit once per week, hypertension, and family history of DM were identified as significantly associated factors with DM.

Pieces of evidences from the National NCDs STEPS Survey in Ethiopia in 2015 reported a prevalence of 3.2% [21], the prevalence of previously undiagnosed DM found in this study 3.9% is congruent to the above evidence. This result is also supported by studies conducted in different settings; Hosanna, Ethiopia, 5.7% [17], Northwest Nigeria, 4.3% [22], Southwest Ethiopia, 3.1% [23], Addis Ababa (2.6%) [7] and Rural Sidama, Ethiopia (1.9%) [24]. However, the observed magnitude is lower than the findings of previous similar studies done in East Gojjam, Ethiopia (11.5%) [18], Southwest Ethiopia (6.5%) [15], Democratic Republic of Congo (11.7%) [25], a global prevalence of 10.5% [2] and Sidama Region, Ethiopia (12.4%) [19], Hawassas City (12.2%) [26] and Accra, Ghana (19.1%) [12]. The observed variation in the magnitude of DM across studies might be due to discrepancies in the setting and design of the study, sample sizes, the socio-demographic characteristics of the study.

Our study found a higher prevalence of DM among females (4.2%) than males (3.8%). Although the observed prevalence among the gender is not statistically different, the study result is inconsistent with studies result that reported a lower prevalence of DM among females than males [21,23]. The observed difference could be explained by the difference in the proportion of the sample size in between the group. Similarly, a higher prevalence (17.5%) of DM was found among study participants who had a family history of DM, who had HTN (13.6%), and participants who had ever smoked a cigarette (10.5%). This finding is consonant with a study reported from Hawassa Zuria District, Southern Ethiopia [19], Northwest Nigeria [22], Mizan Aman town, Southwest Ethiopia [15] where the higher magnitude of DM was observed among study participants who had HTN and ever smoked cigarette. This similarity might be due to the reason that type 1 diabetes has strongly appeared in those individuals who have a family history of DM.

Diabetes estimates for 2021 showed an increasing prevalence of diabetes by age [2]. The finding of this study is supportive of the above evidence, where study participants’ age <35 years had a 79% reduced chance of contracting DM compared to participants with an age of ≥35 years. This finding is consonant to different studies conducted nationally and globally: Southern Ethiopia [24], East Gojjam [18], Mecha District, West Gojjam Zone, Northwest Ethiopia [27], Gilgel Gibe, Southwest Ethiopia [28], Hawassa City, South Ethiopia [26], [National NCDs STEPS Survey in Ethiopia in 2015 [21], Democratic Republic of Congo [25], Accra, Ghana [12], Northern Sudan [20], Hawassa Zuria District [19], Northwest Nigeria[22], Jimma Town, Southwest Ethiopia, [23]. The similarities might be because as age increases the cumulative effect of early life exposure to biological, social, and behavioral determinants of diabetes also increased [13].

Evidence suggested that excessive salt consumption increases the risk of developing type-2 DM through a direct effect on insulin resistance, or through promoting high blood pressure and weight gain [29]. The finding of our study supports the above evidence, where participants who consumed excess salt were fourteen times more likely to experience DM as compared to their counterparts. This is consistent with the fining of different studies conducted both in national and abroad [29–31]. Moreover, in Ethiopia, the findings from the Global Burden of Disease study of 2013 found that high intake of sodium was the most important dietary factor [14]. Likewise, study participants who have hypertension were twenty-nine times more likely of having DM compared to their counterparts. This is similar with different studies conducted nationally [15,19,24,32]. The possible reason might be since in DM there is an increased vascular reactivity to various vasoconstrictors and increased total exchangeable body sodium, which in turn increases the risk of overweight [31].

On the other hand, our study found that study participants who had consumed vegetables and fruits once per week were twenty-three times more likely of developing DM as compared to participants who had consumed vegetables and fruits more than three times per week. This is consistent with other similar studies conducted in Mecha District, West Gojjam Zone, Northwest Ethiopia [27], and China [33]. Similarly, a meta-analysis result showed that a higher intake of fruit and vegetables is associated with a lower risk of type 2 diabetes, however, the mechanisms by which fruit and vegetables could reduce the risk of type 2 DM had not been quite expounded [34]. Another finding from the Global Burden of Disease study of 2013 in Ethiopia reported that low intake of fruits and vegetables were the most important dietary factors for chronic diseases including type-2 DM [14].

Lastly, participants who had a family history of DM were nine times more chance of contracting DM compared to their counterparts. This is congruent with a study conducted to investigate the association between different family history risk categories and the prevalence of diabetes among the Chinese population showed that the risk of DM was higher among study participants who had a family history of DM [35]. Similarly, studies conducted in different places in Ethiopia also supported our result [7,18,32]. The observed similarity might be because attributed to the genetics of diabetes passed from parents and also attributed to shared behavioral risk factors among the families.

### Strength and limitation of the study

This study may have underestimated the prevalence of undiagnosed diabetes because of sampling from the governmental employees; hence this might underestimate the magnitude of undiagnosed cases of diabetes. In addition, alcohol and smoking were also measured based on whether the respondents had ever been exposed providing a crude measure that may not reflect the real exposures. Furthermore, type-1 and type-2 diabetes have not been differentiated despite the varying prevalence and risk factors among old adults.

### Conclusion

A comparable prevalence of 3.9% of previously undiagnosed DM was found by this study. Being old, excess salt consumption, intake of vegetables & fruit once per week, hypertension, and family history of DM were identified as significantly associated factors with DM. The zonal Health department should enhance and strengthen the provision of health education programs and counseling about nutrition, weight control, and appropriate physical activity and advised the communities for mass screening for diabetes.
